# Clinical Treatise on Inebriety

**Published:** 1911-07

**Authors:** 


**Clinical Treatise on Inebriety.** By **T. D. Crothers,** M.D., Superintendent of Walnut Lodge Hospital, Hartford; Editor, Journal of Inebriety. 8 mo, pp. 365. Cincinnati: Harvey Publishing Co. (Cloth, $3.00.)

From time immemorial mankind has always looked upon the inebriate as an offender of society,—a criminal. With the scientific study of crime and its causes, alcoholism has at last been put in its proper category as a disease and only the fanatic and ignorant still look upon it as a violation of the social law.

Among those who stand in the front rank of the investigators of the subject, none other in America, at least, has given more thought and attention nor has had better opportunities for the study of the alcoholic and the drug habitué than the author of this work. For upwards of thirty-five years, he has treated inebriates in and out of his institution. He has lectured and written on the subject, investigated it in all of its phases and as a result of his vast experience gives us a book that is well worth the careful consideration and study of physicians and the lay public.

Paradoxical though it may seem the average practitioner, even in this age of enlightenment, is still ignorant of the proper status of inebriety and oftentimes treats the case as unscientifically as it can be cared for.

This book is worthy of careful perusal by the specialist as well as by the general practitioner. It takes up the drink problem insofar as it concerns the inebriate from all of its aspects—its physiology, psychology, therapy and criminology. There are no far-fetched or fantastic theories stated therein, everything being discussed in a practical manner and presented in a clear and yet forcible manner.

Treatment takes up about a third of the work, the chief points considered being the management of a case both in and out of an institution, the colony treatment of chronic alcoholics, alcoholic psychoses and alcoholic neuroses. L. K.

